# Effects of Coffee Intake on Dyslipidemia Risk According to Genetic Variants in the *ADORA* Gene Family among Korean Adults

**DOI:** 10.3390/nu12020493

**Published:** 2020-02-14

**Authors:** Jihee Han, Jinyoung Shon, Ji-Yun Hwang, Yoon Jung Park

**Affiliations:** 1 Department of Nutritional Science and Food Management, Ewha Womans University, Seoul 03760, Korea; 2 Department of Foodservice Management and Nutrition, Sangmyung University, Seoul 03016, Korea; jiyunhk@smu.ac.kr

**Keywords:** Adenosine receptors, genetic variants, coffee, dyslipidemia, Korean Genome and Epidemiology Study (KoGES)

## Abstract

Current evidence on the effects of coffee intake on cardiovascular diseases is not consistent, in part contributed by the genetic variability of the study subjects. While adenosine receptors (ADORAs) are involved in caffeine signaling, it remains unknown how genetic variations at the *ADORA* loci correlate the coffee intake with cardiovascular diseases. The present study examined the associations of coffee intake with dyslipidemia risk depending on genetic variants in the *ADORA* gene family. The study involved a population-based cohort of 4898 Korean subjects. Consumption of more than or equal to a cup of coffee per day was associated with lower dyslipidemia risk in females carrying the *ADORA2B* minor allele rs2779212 (OR: 0.645, 95% CI: 0.506–0.823), but not in those with the major allele. At the *ADORA2A* locus, male subjects with the minor allele of rs5760423 showed instead an increased risk of dyslipidemia when consuming more than or equal to a cup of coffee per day (OR: 1.352, 95% CI: 1.014–1.802). The effect of coffee intake on dyslipidemia risk differs depending on genetic variants at the *ADORA* loci in a sex-specific manner. Our study suggests that a dietary guideline for coffee intake in the prevention and management of dyslipidemia ought to consider ADORA-related biomarkers carefully.

## 1. Introduction

While coffee consumption has increased globally, the research on biological function and effects of coffee intake remain controversial [1]. A meta-analysis of randomized controlled trials stated that coffee intake changed blood lipid profiles, including increase of cholesterol and triglyceride (TG) [2], which are clinical indicators for cardiovascular disease risk. On the contrary, other meta-analysis on observational and interventional studies suggested that high coffee consumption was associated with reduced risks of cardiovascular disease and mortality [3,4,5].

Genetic variation has been suggested as one of the main reasons why individuals respond differently to coffee intake [6]. Focused studies have been conducted on the ADORA locus and its genetic variants because their expression is antagonized by caffeine and, in turn, play a role in transmitting the effects of coffee intake throughout the body [7].

Studies investigating the association between coffee intake and the ADORA gene family have focused on neuronal effects such as habitual coffee intake [8], arousal [9], sleep disorders [10], and anxiety [11], or on blood pressure [6]. The *ADORA* gene family, composed of *ADORA1*, *ADORA2A*, *ADORA2B*, and *ADORA3*, are differently expressed in a tissue-specific manner and show unique properties in regulating multiple physiological statuses [7]. A recent review highlighted that the *ADORA* gene members are modulators of lipid availability [12]. The physiological role of the ADORAs has been reported to be associated with lipid-related diseases, including cardiovascular disease [13], coronary blood flow [14], chronic heart failure [15], atherosclerosis, and dyslipidemia [16].

While the association of the *ADORA* gene family between coffee intake and multiple lipid-related diseases have been investigated in several studies, we hypothesized that the discrepancies in the findings of coffee intake with regard to dyslipidemia might be explained by genetic variants, which henceforth motivated this study.

## 2. Materials and Methods

### 2.1. Study Population

This study was conducted with a local community-based cohort emanating from the Korean population-based cohorts of the Korean Genome and Epidemiology Study [17]. The local community-based cohort included residents living in rural Ansung and urban Ansan since 2001. All subjects provided informed consent at baseline. The cohort was examined by follow-up surveys every two years, and the eighth follow-up survey was performed in 2018. This study used data from the second follow-up survey conducted from 2005 to 2006.

**Figure 1:**
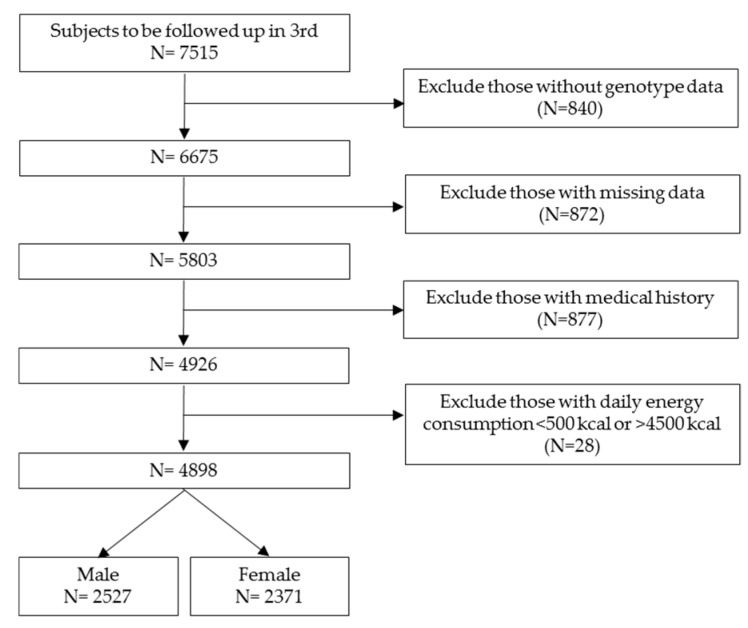
A flow chart of subject selection.

From a total of 7515 subjects, aged 43–74 years, we excluded 2617 subjects with missing data, those with daily energy consumption <500 kcal or >4500 kcal, those with previous history and presence of diabetes, renal disease, thyroid disease, cardiovascular disease, cancer, hysterectomy, and ovariectomy, and those who received medications for those diseases. Finally, 2527 male and 2371 female subjects were included in this study (Figure 1).

Dyslipidemia was defined as dyslipidemia diagnosis, related drug use, and abnormal lipid profile (low-density lipoprotein-cholesterol ≥ 160 mg/dL, TG ≥ 200 mg/dL, total cholesterol (TC) ≥ 240 mg/dL, and high-density lipoprotein-cholesterol <40 mg/dL). Blood pressure was the average of three measurements with five minutes interval, taken in the morning after 10 min of rest in sitting position. Coffee intake was assessed using the food-frequency questionnaire. Depending on the amounts of coffee intake per week, the subjects were divided into those who consumed less than one cup of coffee per day (low coffee intake group) and those who consumed more than or equal to one cup of coffee per day (high coffee intake group). A cup was estimated as much as 150 mL. This study was approved by the Institutional Review Board of Ewha Womans University, Seoul, Korea (IRB No. 129-17).

### 2.2. Genotyping and Analysis of Single Nucleotide Polymorphisms

**Figure 2:**
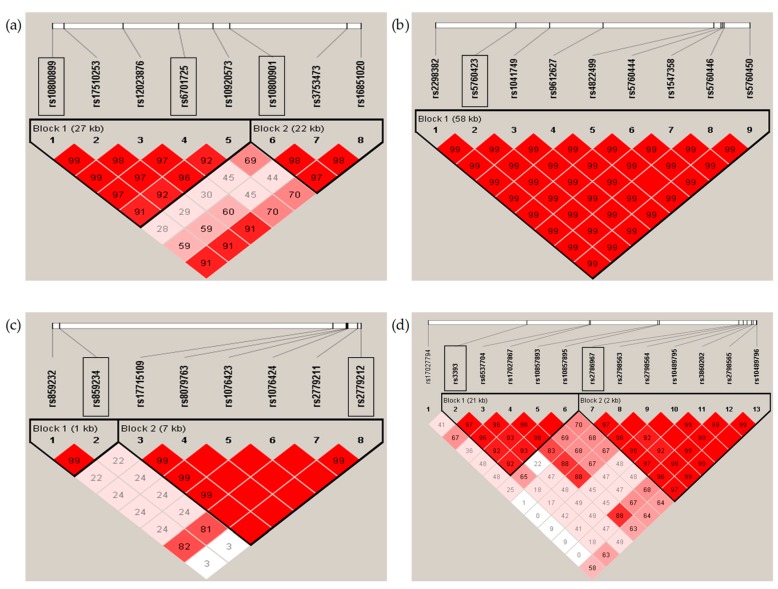
Linkage Disequilibrium (LD) block of genetic variants in the *Adenosine Receptor* (*ADORA*) gene family. The LD blocks are for variants in (**a**) *ADORA1*, (**b**) *ADORA2A*, (**c**) *ADORA2B*, and (**d**) *ADORA3* loci, respectively. Square boxes indicate SNPs used for further analysis.

Genomic DNA was collected from peripheral blood samples of the subjects and genotyped on Affymetrix Genome-Wide Human SNP Array 5.0, as previously described [18]. Among SNPs in four loci encoding ADORAs, 79 SNPs were included in the platform. The missing call rate (>5%), deviation from Hardy–Weinberg equilibrium (HWE) (*p* < 1 × 10^6^), or minor allele frequency (*p* < 0.05) was used to eliminate 38 inadequate SNPs in the sample population. Among the remaining 38 SNPs, 30 SNPs were removed due to high levels of pairwise linkage disequilibrium (LD) (Figure 2). Finally, eight of the 79 SNPs were used for further analysis (Table 1).

### 2.3. Statistical Analysis

Statistical analyses were performed using the SAS program (SAS 9.4, 2016, SAS Institute, Cary, NC, USA). Data are presented as mean with standard deviation (SD). The numbers in brackets are percentages in the column. To compare differences between groups, we used Student’s *t*-test for numeric variables after log transformation and the chi-square test for categorical variables. Odds ratio (OR) and 95% confidence interval (CI) were calculated to evaluate te associations among variables by using logistic regression analysis. OR and 95% CI were adjusted with the following confounders: age, marital status, income, education, smoking behavior, energy intake, systolic blood pressure, and body mass index (BMI) in male subjects and age, income, education, drinking behavior, smoking behavior, energy intake, BMI, menopause, female hormone treatment, and hypertension in female subjects. The *p*-value for the interaction between genetics and coffee intake was calculated. Findings were considered significant at *p* < 0.05. Calculation of allele frequencies and HWE and variant pruning based on LD were conducted using the software package PLINK v1.09 [19]. Pairwise LD blocks of genetic variants in the ADORA gene family were produced by Haploview 4.2 [20]. After testing different genetic models, including dominant, recessive, and additive models, the recessive model was selected for this study.

## 3. Results

### 3.1. Basic Characteristics Depending on Coffee Consumption

Table 2 shows the basic characteristics of the subjects according to sex and the amount of coffee intake. In both male and female subjects, those in the high coffee intake group were younger, had a higher income, had a longer duration of education, and were more frequently current smokers when compared with the findings in the low coffee intake group. Energy consumption was higher in the high coffee intake group than in the low coffee intake group. Additionally, the high coffee intake group showed a significantly lower consumption of sugar and the proportion of carbohydrates in energy distribution when compared with the findings in the low coffee intake group.

In contrast, the mean intake of fat was higher in the high coffee intake group than in the low coffee intake group, and the finding was in accordance with an increased ratio of energy distribution. Systolic blood pressure was lower in the high coffee intake group than in the low coffee intake group. However, hip circumference, height, and weight were higher in the high coffee intake group than in the low coffee intake group. Among both male and female subjects, the TC level was higher in the high coffee intake group than in the low coffee intake group. However, the TG level was higher among male subjects and lower among female subjects in the high coffee intake group than in the low coffee intake group. Among female subjects, the prevalence of hypertension and menopause were lower in the high coffee intake group than in the low coffee intake group.

### 3.2. Association of Coffee Intake with the Risk of Dyslipidemia

We next examined the effect of coffee intake on dyslipidemia risk. There was an inverse correlation between coffee intake and the prevalence of dyslipidemia in female subjects (OR: 0.768, 95% CI: 0.645–0.914, *p* = 0.0030) but not in male subjects (*p* = 0.2635) after adjusting for confounders (Table 3).

### 3.3. Effects of Coffee Intake on the Risk of Dyslipidemia Depending on ADORA Gene Family

Finally, we performed a logistic regression analysis to confirm the genetic effect of the *ADORA* gene family on the association between coffee intake and dyslipidemia risk (Table 4 and Table 5). Interestingly, among female subjects, a favorable effect of consuming more coffee on dyslipidemia risk showed only those with the minor alleles of *ADORA1* rs10800901 (OR: 0.727, 95% CI: 0.560–0.944, *p* = 0.0168), and *ADORA2B* rs2779212 (OR: 0.645, 95% CI: 0.506–0.823, *p* = 0.0004) and the major alleles of *ADORA3* rs2786967 (OR: 0.818, 95% CI: 0.676–0.989, *p* = 0.0384), but not in those with alternative alleles. Among male subjects, there was instead an increased dyslipidemia risk on consuming more coffee carrying the minor alleles of *ADORA2A* rs57604223 (OR: 1.352, 95% CI: 1.014–1.802, *p* = 0.0402). Male subjects with the minor allele of *ADORA3A* rs3393 also showed lower risk on dyslipidemia (Table S1), and the favorable effects did not occur when they consumed more coffee. Overall, these results indicate that the effect of coffee intake on dyslipidemia risk depends on genetic variants in the *ADORA* gene family in a sex-specific manner.

## 4. Discussion

The present study aimed to investigate whether genetic variants in the *ADORA* gene family influence the effect of coffee intake on dyslipidemia risk. Coffee intake was associated with decreased dyslipidemia risk in female subjects but not in male subjects. Furthermore, with regard to the genetic effect on the association, the favorable effect of coffee intake among female subjects depends on a subset of genetic variants in *ADORA* gene family. The risk of dyslipidemia was also increased among male subjects in the high coffee intake group based on genetic variation of the *ADORA* gene family, indicating that a subset of genetic variants in the *ADORA* gene family modulates the effect of coffee intake on dyslipidemia risk in a sex-specific manner.

The *ADORA* gene family has been reported to play a role in regulating the lipid profile [12]. For instance, ADORA1 deficiency in ApoE KO mice was associated with increased plasma lipid levels [22], and *ADORA2B* knockout mice showed increased TG and TC levels compared to the wildtype [23]. Disturbed lipid levels via modulation of *ADORA2B* also influenced the development of dyslipidemia and atherosclerosis, known risk factors of cardiovascular mortality [16]. ADORA2B also showed a close relationship with cholesterol regulation by formation of foam cells and inflammation, which are mediator of cardiovascular disease [13,16]. In addition to the functional relevance of the ADORAs in blood lipid profiles and lipid-related chronic diseases, a genetic variant of *ADORA2A* showed association with the severity of chronic heart failure in Asians [15]. The evidence proposed that variations in the ADORA gene family might influence lipid regulation and cardiovascular disease. We also observed a subset of genetic variants in the *ADORA* gene family associated with the risk of dyslipidemia (Table S1).

Despite the interesting finding of an association between the *ADORA* gene family and dyslipidemia itself, the novelty here is that the ADORAs modulates the effect of coffee intake on dyslipidemia. A meta-analysis showed coffee intake increase blood lipid level [2], but not all of the included studies satisfied the result [3,4,5]. We identified different effects of coffee intake in the risk of dyslipidemia linked to their genetic variants in the *ADORA* gene family. Even though there was no association between coffee intake and dyslipidemia in male, we confirmed the increased risk of dyslipidemia when subjects with the minor allele of rs5760423 in *ADORA2A* consumed more than one cup of coffee. While we did not experimentally examine the association, instead only focusing on the association of genetic variants in *ADORA* gene family with coffee intake in dyslipidemia, we identified a subset of genetic variants in the *ADORA* gene family located at regulatory elements which could play a role as eQTLs influencing gene expression in various tissues [24] (Table 1). Indeed, a recent study suggested that genetic variation could contribute to altered gene expression by changing epigenetic enhancer activity, which, in turn, is linked to five different vascular diseases [25]. Given the previous reports, genetic variants in ADORA gene family might modify gene expression through epigenetic regulation, possibly modulating the lipid profile and the effect of coffee intake in dyslipidemia pathogenesis. Further studies are needed to elucidate their possible functional mechanisms.

We also observed a favorable association between coffee intake and the prevalence of dyslipidemia in female subjects but not in male subjects. Inconsistent results of coffee intake between male and female individuals [26,27], including a Korean population [28], obscure the view. Female individuals responded favorably to coffee concerning cardiovascular health. It has been proposed that the female sex hormone estrogen plays a role in the sensitivity of female individuals to the effects of coffee intake [29]. Estrogen is synthesized from cholesterol in the ovary, and it influences lipid metabolism by increasing lipoprotein lipase activity and is directly interacting with specific estrogen receptors in the adipose tissue. Thus, susceptibility to cardiovascular diseases is lower in premenopausal women than in men of the same age and postmenopausal women [30]. A previous finding that coffee intake increases the concentration of estrogen in Asian female individuals could explain the sex-specific differences in the effect of coffee intake on dyslipidemia [29].

The most interesting of our findings is that increased coffee intake had beneficial effects in female subjects but harmful effects in male subjects significantly associated with a subset of genetic variants in the *ADORA* gene family. This could suggest that the response to environmental factors of the ADORAs differs according to sex. Several previous studies showed different influences of environmental factors related to the *ADORA* genotypes depending on sex. Treatment with the ADORA antagonist ATL444 was shown to have a preventive effect on cocaine addiction in male individuals but not in female individuals [31]. Additionally, locomotor activity in response to administration of caffeine was higher in male WT mice than in male *ADORA2A* knockout mice, however, this difference was not noted in female mice. Although the reason why the *ADORA* genotype causes a difference in the environmental response depending on sex is not known, a possible explanation may be that dopamine receptor 2 (D2) and the ADORA2A system are more sensitive in female than in male individuals [32,33]. Dopamine signaling has been suggested as a therapeutic target of dyslipidemia, showing cardioprotective effects [32]. Caffeine treatment has been shown to increase the expression of D2 protein in female but not in male individuals [33]. Based on our data, we suggest that not only do D2 but also the ADORAs modulate the environmental response of the sex-specific physiological mechanism.

We found a novel gene-environment interaction of the *ADORA* genetic variants and coffee intake on dyslipidemia in a Korean population. However, further, larger studies are warranted to replicate the findings. In addition, while our study did not consider how subjects consumed coffee and how much caffeine was present owing to the limited information in the original cohort, we appreciate the importance of further studies including those parameters. Although it has been reported that the addition of milk, the type of coffee bean, and the type of roasting method do not alter antioxidant activity [34], it may be important to consider these factors to perform an in-depth analysis. Lastly, our analysis did not consider physical activity as a confounding factor, although it has been shown to influence blood lipid profiles [35,36]. Additional confounding factors, such as physical activity, may need to be considered for further analysis.

## 5. Conclusions

This study demonstrated that a subset of genetic variants in the *ADORA* gene family influences the association between coffee intake and dyslipidemia risk in a sex-specific manner. As a first study to elucidate the effect of coffee intake on dyslipidemia risk in terms of genetic variability in the *ADORA* gene family, important avenues of detailed research are available. This includes deep understanding of the functional mechanisms on the genetic variants in the *ADORA* gene family in response to coffee intake, potentially aiding prevention and management of dyslipidemia among individuals vulnerable to the disease.

## Figures and Tables

**Table 1:** The list of selected SNPs in the *Adenosine Receptor* (*ADORA*) gene family.

Gene	SNP ID	Chr	Physical Position	Location	Regulatory Element	Alleles ^1^	MAF	HWE
*ADORA1*	rs10800899	1	203081125	intron		A/G	0.1607	0.2697
	rs6701725		203102728	intron		A/G	0.1735	0.1381
	rs10800901		203111304	intron		G/A	0.4486	0.7471
*ADORA2A*	rs5760423	22	24840118	intron		T/G	0.4439	0.2536
*ADORA2B*	rs17715109	17	15869557	intron	H3K4me1, Dnase1	T/G	0.0517	0.5145
	rs2779212		15876655	intron	H3K4me1, eQTL	C/T	0.2287	0.4510
*ADORA3*	rs3393	1	112042149	UTR-3	Dnase1	T/C	0.4457	0.7306
	rs2786967		112075948	intron		G/A	0.8891	0.8767

^1^ Alleles are presented as minor/major alleles. SNP, single nucleotide polymorphism; Chr, chromosome; MAF, minor allele frequency; HWE, Hardy–Weinberg equilibrium.

**Table 2:** Basic Characteristics depending on the amount of coffee intake in male and female ^1^.

	Male			Female		
Coffee	<1 cup/d	≥1 cup/d	*p* ^2^	<1 cup/d	≥1 cup/d	*p* ^2^
	(*n* = 837)	(*n* = 1690)		(*n* = 1112)	(*n* = 1259)	
Age (year)	56.62 ± 8.90	53.95 ± 8.13	<0.0001	57.28 ± 8.75	54.55 ± 8.73	<0.0001
Marriage ^3^						
Married	798 (95.34)	1639 (96.98)	0.0361	939 (84.44)	1071 (85.07)	0.6725
Monthly income (×10^4^ KRW)						
Low (<100)	251 (29.99)	351 (20.77)	<0.0001	524 (47.12)	468 (37.17)	<0.0001
Medium (100–199)	199 (23.78)	356 (21.07)		268 (24.10)	254 (20.17)	
High (≥200)	387 (46.24)	983 (58.17)		320 (28.78)	537 (42.65)	
Education (year)						
Low (0–6)	217 (25.93)	313 (18.52)	<0.0001	619 (55.67)	508 (40.35)	<0.0001
Medium ((7–9)	171 (20.43)	348 (20.59)		222 (19.96)	264 (20.97)	
High (≥10)	449 (53.64)	1029 (60.89)		271 (24.37)	487 (36.68)	
Alcohol drinking behavior						
Never	154 (18.40)	335 (19.82)	0.6875	869 (78.15)	817 (64.89)	<0.0001
Former	70 (8.36)	136 (8.05)		22 (1.98)	19 (1.51)	
Current	613 (73.24)	1219 (72.13)		221 (19.87)	423 (33.60)	
Alcohol intake (g/day) ^4^	25.77 ± 36.07	25.00 ± 31.27	0.5934	3.99 ± 8.89	4.88 ± 11.01	<0.0001
Smoking behavior						
Never	287 (34.29)	346 (20.47)	<0.0001	1094 (98.38)	1219 (96.82)	0.0489
Former	304 (36.32)	642 (37.99)		5 (0.45)	12 (0.95)	
Current	246 (29.39)	702 (41.54)		13 (1.17)	28 (2.22)	
Tobacco consumption (pack/years) ^5^	16.61 ± 14.57	20.25 ± 18.42	<0.0001	7.38 ± 6.04	7.42 ± 8.31	0.0289
Nutrient intakes						
Energy (Kcal)	1,772.21 ± 510.35	1984.88 ± 527.16	<0.0001	1594.52 ± 497.34	1753.07 ± 532.77	<0.0001
Sugar (g/per 1000 Kcal)	180.35 ± 16.89	177.75 ± 14.54	<0.0001	186.28 ± 16.97	182.06 ± 15.48	<0.0001
Fat (g/per 1000 Kcal)	14.72 ± 5.89	16.44 ± 5.13	<0.0001	12.68 ± 5.89	14.85 ± 5.44	<0.0001
Protein (g/per 1000 Kcal)	32.52 ± 5.93	32.48 ± 5.13	0.8755	31.88 ± 5.30	32.44 ± 5.97	0.0269
Energy distribution (%)						
Carbohydrate	73.35 ± 7.20	71.93 ± 6.13	<0.0001	75.56 ± 7.32	73.47 ± 6.63	<0.0001
Fat	13.44 ± 5.31	14.94 ± 4.61	<0.0001	11.53 ± 5.29	13.45 ± 4.85	<0.0001
Protein	13.21 ± 2.35	13.13 ± 2.03	0.4204	12.91 ± 2.45	13.07 ± 2.33	0.0946
SBP (mmHg)	117.73 ± 15.80	116.12 ± 14.87	0.0147	116.00 ± 17.26	113.95 ± 16.84	0.0030
DBP (mmHg)	79.74 ± 10.11	79.18 ± 10.10	0.1838	76.61 ± 10.37	75.58 ± 10.53	0.0131
Waist circumference (cm)	84.22 ± 7.76	84.77 ± 7.46	0.0692	83.98 ± 9.68	82.76 ± 9.45	0.0022
Hip circumference (cm)	90.90 ± 5.23	92.32 ± 5.21	<0.0001	91.13 ± 5.35	92.18 ± 5.25	<0.0001
Height (cm)	166.32 ± 5.95	167.15 ± 5.83	0.0008	153.23 ± 5.84	153.94 ± 5.63	0.0026
Weight (kg)	65.71 ± 9.27	68.04 ± 9.57	<0.0001	57.55 ± 8.45	58.76 ± 8.03	0.0004
BMI (kg/m^2^) ^6^						
Underweight (<18.5)	26 (3.11)	29 (1.72)	0.0003	18 (1.62)	15 (1.19)	0.0739
Normal (18.5–22.9)	290 (34.65)	502 (29.70)		364 (32.73)	356 (28.28)	
Overweight (23–24.9)	253 (30.23)	485 (28.70)		284 (25.54)	356 (28.28)	
Obese (≥25)	268 (32.02)	674 (39.88)		446 (40.11)	532 (42.26)	
HbA1C (%)	5.41 ± 0.41	5.41 ± 0.39	0.9575	5.47 ± 0.40	5.44 ± 0.40	0.0628
Total cholesterol (mg/dL)	183.12 ± 34.42	191.06 ± 32.64	<0.0001	193.01 ± 34.56	196.65 ± 33.53	0.0060
HDL-Cholesterol (mg/dL)	43.90 ± 10.86	43.04 ± 10.24	0.0614	44.83 ± 9.77	46.75 ± 10.16	<0.0001
Triglyceride (mg/dL)	144.81 ± 107.99	150.9 ± 117.12	0.0385	124.89 ± 69.71	116.24 ± 66.91	0.0001
Menopause				789 (70.95)	712 (56.55)	<0.0001
Female hormone treatment				25 (2.25)	34 (2.70)	0.4804
Hypertension ^7^	259 (30.94)	482 (28.52)	0.2079	367 (33.00)	333 (26.45)	0.0005

^1^ Data are presented as the means ± SDs or n (%). ^2^ Statistical significance was calculated with Student’s *t*-tests for continuous variables after log transformation and chi-square tests for categorical variables. ^3^ Married included married and cohabitation. ^4^ Data were collected from current alcohol consumers without missing responders; *n* = 610, 1216 in male and 220, 421 in female, respectively. ^5^ Data were collected from former and current smokers without missing responders; *n* = 189, 372 in male and 13, 24 in female, respectively. ^6^ Degree of obesity was categorized into four stages according to the criterion of World Health Organization (WHO) Asia-Pacific Area [21]. ^7^ Subjects with diagnosis in medical history.

**Table 3:** Associations between coffee intake and the risk of dyslipidemia.

Coffee	Male				Female			
	Healthy	DLP	Adjusted Model ^1^		Healthy	DLP	Adjusted Model ^2^	
	(*n* = 1215)	(*n* = 1312)	OR (95% CI)	*p* ^3^	(*n* = 1330)	(*n* = 1041)	OR (95% CI)	*p* ^3^
<1 cup/d	427 (35.14)	410 (31.25)	1		571 (42.93)	541 (51.97)	1	
≥1 cup/d	788 (64.86)	902 (68.75)	1.107 (0.926–1.323)	0.2635	759 (57.07)	500 (48.03)	0.768 (0.645–0.914)	0.0030

^1^ Adjusted for age, marital status, income, education, smoking behavior, energy intake, systolic blood pressure, and BMI. ^2^ Adjusted for age, income, education, drinking and smoking behavior, energy intake, BMI, menopause, treatment of female hormone, and hypertension. ^3^ Odds ratio (OR), 95% confidence interval (95% CI), and statistical significance were calculated with logistic regression analysis. DLP, dyslipidemia.

**Table 4:** Risk of dyslipidemia depending on the coffee intake and genotype in ADORA gene family in male.

Genes	Alleles	Coffee Intake	Healthy	Dyslipidemia	Adjusted Model ^1^		
SNPs			(*n* = 1215)	(*n* = 1312)	Odds Ratios (95% CI)	*p*	*p* ^2^
***ADORA1***							
rs10800899	GG	<1 cup/d	299 (24.61)	274 (20.88)	1		0.8839
		≥1 cup/d	541 (44.53)	641 (48.86)	1.176 (0.952–1.453)	0.1327	
	AG/AA	<1 cup/d	128 (10.53)	136 (10.37)	1.103 (0.816–1.493)	0.5231	
		≥1 cup/d	247 (20.33)	261 (19.89)	1.064 (0.828–1.368)	0.6267	
rs6701725	GG	<1 cup/d	294 (24.20)	277 (21.11)	1		0.3714
		≥1 cup/d	534 (43.95)	625 (47.64)	1.127 (0.911–1.394)	0.2723	
	AG/AA	<1 cup/d	133 (10.95)	133 (10.14)	0.991 (0.733–1.341)	0.9542	
		≥1 cup/d	254 (20.91)	277 (21.11)	1.056 (0.823–1.355)	0.6691	
rs10800901	AA	<1 cup/d	131 (10.78)	118 (8.99)	1		0.6906
		≥1 cup/d	246 (20.25)	265 (20.20)	1.115 (0.812–1.532)	0.5003	
	GA/GG	<1 cup/d	296 (24.36)	292 (22.26)	1.132 (0.833–1.540)	0.4277	
		≥1 cup/d	542 (44.61)	637 (48.55)	1.250 (0.938–1.665)	0.1276	
***ADORA2A***							
rs5760423	GG	<1 cup/d	138 (11.36)	111 (8.46)	1		0.8317
		≥1 cup/d	264 (21.73)	283 (21.57)	1.246 (0.910–1.706)	0.1699	
	TG/TT	<1 cup/d	289 (23.79)	299 (22.79)	1.282 (0.942–1.744)	0.1139	
		≥1 cup/d	524 (43.13)	619 (47.18)	1.352 (1.014–1.802)	0.0402	
***ADORA2B***							
rs17715109	GG	<1 cup/d	381 (31.36)	360 (27.44)	1		0.8732
		≥1 cup/d	712 (58.60)	817 (62.27)	1.130 (0.936–1.365)	0.2020	
	TG/TT	<1 cup/d	46 (3.79)	50 (3.81)	1.149 (0.738–1.786)	0.5389	
		≥1 cup/d	76 (6.26)	85 (6.48)	1.076 (0.753–1.536)	0.6878	
rs2779212	TT	<1 cup/d	252 (20.74)	226 (17.23)	1		0.0336
		≥1 cup/d	494 (40.66)	555 (42.30)	1.187 (0.943–1.494)	0.1433	
	CT/CC	<1 cup/d	175 (14.40)	184 (14.02)	1.155 (0.870–1.534)	0.3182	
		≥1 cup/d	294 (24.20)	347 (26.45)	1.165 (0.906–1.497)	0.2345	
***ADORA3***							
rs3393	CC	<1 cup/d	122 (10.04)	147 (11.20)	1		0.5917
		≥1 cup/d	228 (18.77)	284 (21.65)	0.990 (0.725–1.350)	0.9477	
	TC/TT	<1 cup/d	305 (25.10)	263 (20.05)	0.731 (0.541– 0.989)	0.0423	
		≥1 cup/d	560 (46.09)	618 (47.10)	0.856 (0.647–1.133)	0.2767	
rs2786967	AA	<1 cup/d	361 (29.71)	357 (27.21)	1		0.2144
		≥1 cup/d	676 (55.64)	752 (57.32)	1.036 (0.856– 1.255)	0.7152	
	GA/GG	<1 cup/d	66 (5.43)	53 (4.04)	0.771 (0.514– 1.155)	0.2066	
		≥1 cup/d	112 (9.22)	150 (11.43)	1.257 (0.933–1.694)	0.1324	

^1^ Adjusted for age, marital status, income, education, smoking behavior, energy intake, systolic blood pressure, and BMI. ^2^ *p* for interaction.

**Table 5:** Risk of dyslipidemia depending on the coffee intake and genotype in ADORA gene family in female.

Genes	Alleles	Coffee Intake	Healthy	Dyslipidemia	Adjusted Model ^1^		
SNPs			(n = 1215)	(n = 1312)	Odds ratios (95% CI)	*p*	*p* ^2^
***ADORA1***							
rs10800899	GG	<1 cup/d	392 (29.47)	374 (35.93)	1		0.2012
		≥1 cup/d	537 (40.38)	366 (35.16)	0.789 (0.643–0.968)	0.0233	
	AG/AA	<1 cup/d	179 (13.46)	167 (16.04)	0.989 (0.761–1.286)	0.9356	
		≥1 cup/d	222 (16.69)	134 (12.87)	0.705 (0.537–0.924)	0.0115	
rs6701725	GG	<1 cup/d	393 (29.55)	367 (35.25)	1		0.3393
		≥1 cup/d	520 (37.10)	346 (33.24)	0.784 (0.636–0.966)	0.0225	
	AG/AA	<1 cup/d	178 (13.38)	174 (16.71)	1.031 (0.794–1.339)	0.8196	
		≥1 cup/d	239 (17.97)	154 (14.79)	0.758 (0.585–0.982)	0.0356	
rs10800901	AA	<1 cup/d	180 (13.53)	172 (16.52)	1		0.3401
		≥1 cup/d	210 (15.79)	160 (15.37)	0.891 (0.656–1.210)	0.4956	
	GA/GG	<1 cup/d	391 (29.40)	369 (35.45)	1.008 (0.777–1.309)	0.9504	
		≥1 cup/d	549 (41.28)	340 (32.66)	0.727 (0.560–0.944)	0.0168	
***ADORA2A***							
rs5760423	GG	<1 cup/d	194 (14.59)	173 (16.62)	1		0.3188
		≥1 cup/d	224 (16.84)	149 (14.31)	0.836 (0.617–1.134)	0.2497	
	TG/TT	<1 cup/d	377 (28.35)	368 (35.35)	1.124 (0.869–1.456)	0.3736	
		≥1 cup/d	535 (40.23)	351 (33.72)	0.829 (0.641–1.073)	0.1539	
***ADORA2B***							
rs17715109	GG	<1 cup/d	508 (38.20)	486 (46.69)	1		0.7766
		≥1 cup/d	671 (50.45)	461 (44.28)	0.795 (0.662–0.956)	0.0146	
	TG/TT	<1 cup/d	63 (4.74)	55 (5.28)	0.903 (0.608–1.339)	0.6107	
		≥1 cup/d	88 (6.62)	39 (3.75)	0.500 (0.332–0.754)	0.0009	
rs2779212	TT	<1 cup/d	343 (25.79)	335 (32.18)	1		0.8210
		≥1 cup/d	421 (31.65)	301 (28.91)	0.839 (0.671–1.049)	0.1226	
	CT/CC	<1 cup/d	228 (17.14)	206 (19.79)	0.943 (0.735–1.210)	0.6469	
		≥1 cup/d	338 (25.41)	199 (19.12)	0.645 (0.506–0.823)	0.0004	
***ADORA3***							
rs3393	CC	<1 cup/d	179 (13.46)	163 (15.66)	1		0.9277
		≥1 cup/d	247 (18.57)	157 (15.08)	0.776 (0.572–1.053)	0.1035	
	TC/TT	<1 cup/d	392 (29.47)	378 (36.31)	1.053 (0.809–1.370)	0.7011	
		≥1 cup/d	512 (38.50)	343 (32.95)	0.806 (0.618–1.051)	0.1109	
rs2786967	AA	<1 cup/d	486 (36.54)	444 (42.65)	1		0.0308
		≥1 cup/d	617 (46.39)	411 (39.48)	0.818 (0.676–0.989)	0.0384	
	GA/GG	<1 cup/d	85 (6.39)	97 (9.32)	1.306 (0.939–1.816)	0.1125	
		≥1 cup/d	142 (10.68)	89 (8.55)	0.737 (0.542–1.002)	0.0515	

^1^ Adjusted for age, income, education, drinking and smoking behavior, energy intake, BMI, menopause, treatment of female hormone, Hypertension. ^2^ *p* for interaction.

## Supplementary Materials

The following are available online at https://www.mdpi.com/2072-6643/12/2/493/s1, Table S1. Association between genetic variants in ADORA gene family and the risk of dyslipidemia.
